# Concurrent Validity of Two Inertial Measurement Unit Pipelines for Estimating Lumbar and Thoracic Kinematics During Lifting Tasks

**DOI:** 10.3390/s26092639

**Published:** 2026-04-24

**Authors:** Samantha J. Snyder, Aditi Mannby, Dario Martelli

**Affiliations:** 1 Department of Orthopedics and Sports Medicine, MedStar Health Research Institute, Baltimore, MD 21218, USA; samantha.snyder@medstar.net (S.J.S.); amannby1@jh.edu (A.M.); 2 Department of Biomedical Engineering, Johns Hopkins University, Baltimore, MD 21218, USA

**Keywords:** lift, low back pain, inertial measurement unit, lumbar, thoracic, kinematics

## Abstract

Lumbosacral and thoracolumbar kinematics are key risk factors for lifting-related low back pain, yet their measurement is typically restricted to motion capture laboratories. Inertial measurement units (IMUs) offer the potential to quantify spine kinematics in more naturalistic settings, but the validity of IMU-based processing pipelines relative to optical motion capture (OMC) remains unclear. Nine healthy participants performed stoop, squat, free, and asymmetric lifting tasks while IMU and OMC data were simultaneously collected to evaluate the concurrent validity of two IMU pipelines: the proprietary MVN Analyze pipeline and an OpenSense pipeline using a validated OpenSim biomechanical model for lifting. Joint angles from both pipelines were compared against OMC-derived joint angles calculated using the same validated OpenSim model with one-way repeated-measures statistical parametric mapping (SPM) (*α* = 0.05), Bland–Altman analysis with Limits of Agreement (LoA) and 95% Confidence Intervals (CIs), and Concordance Correlation Coefficients (CCCs) with 95% CIs. Xsens MVN Analyze consistently overestimated flexion-extension at both spinal levels across all lift types (lumbosacral: RMSE ≤ 9.8°, bias ≤ −14.5°, LoA ≤ ±10°; thoracolumbar: RMSE ≤ 5.4°, bias ≤ −8.3°, LoA ≤ ±5°), with SPM confirming significant differences during the lifting and lowering phases of all lifting cycles. In contrast, processing Xsens data with OpenSense using the same biomechanical model as the OMC data yielded excellent agreement with OMC (RMSE ≤ 2.9°, bias ≤ 3°, LoA ≤ ±10°). CCC was poor to moderate, specifically in lateral bending and axial rotation planes, likely reflecting limited between-participant ROM variability. These results suggest that discrepancies are driven primarily by biomechanical model differences rather than sensor or sensor fusion limitations. Ultimately, when paired with an appropriate biomechanical model, XSens sensors show promise for practical field-based assessment of lifting biomechanics, potentially requiring only sensors at the chest and pelvis.

## 1. Introduction

Low back pain is associated with a reduced quality of life and is strongly influenced by work-related factors [1]. Occupations that involve frequent bending and additional weight-bearing activities such as lifting are associated with an increased risk of developing low back pain [2]. Given this association, accurate measurement of trunk kinematics is important to assess the risk of trunk musculoskeletal injuries [3,4,5,6].

Although the trunk is often modeled as a single rigid segment [7,8,9,10,11], evaluating motion at individual spinal levels provides essential information about how different regions contribute to overall trunk movement. Segment-level analysis, particularly at the thoracolumbar (T12/L1) and lumbosacral (L5/S1) joints, also improves understanding of how mechanical loads are distributed across the spine. Indeed, these spinal joint kinematics, along with external forces, can be incorporated into musculoskeletal models implemented in software such as OpenSim version 4.5 [12] to estimate internal joint loading and muscle forces at specific spinal segments. This information is critical for identifying injury mechanisms, characterizing movement strategies during lifting, and quantifying the mechanical demands placed on spinal tissues. Complex full-body thoracolumbar models implemented in OpenSim [12], combined with kinematic constraints, have been validated using optical motion capture (OMC) to produce physiologically plausible spine kinematics during lifting movements [13,14,15,16,17]. Although OMC is considered the gold standard for measuring kinematics, these measurements are typically confined to biomechanical laboratories, making real-life measurement during the workday challenging. Wearable inertial measurement unit (IMU) sensors, which measure angular velocity and linear acceleration of body segments, have grown in popularity as a practical alternative for quantifying joint kinematics outside the laboratory. Their validity for quantifying lower- and upper-limb segment orientations during various activities has been well established [18,19,20,21,22], with joint angle errors generally falling within the clinically acceptable threshold of 5° [23].

Despite this evidence, validation of IMU-based measurement of thoracolumbar and lumbosacral joint kinematics during lifting remains limited. The majority of prior work has focused broadly on rigid body lumbar segment kinematics or modeling the entire trunk as a single rigid segment [7,9,10,11,22,24,25,26]. One exception to this previous research is a study that placed IMU sensors directly over specific spinous processes using ultrasound guidance, resolving relative and global segment angles estimated at individual vertebral levels [27]. However, this approach requires trained personnel and a more complex setup, which may limit its suitability for routine or large-scale applications. Moreover, validation was conducted during spine range-of-motion tasks rather than lifting [27], and given that the accuracy of wearable systems is task-dependent [28], its generalization to occupational contexts remains uncertain. Further investigation using three-dimensional, segment-level measurements during lifting is therefore warranted. To address these challenges, full-body IMU systems such as Xsens MVN Analyze (Xsens MVN Analyze; Xsens Technologies, Enschede, The Netherlands) are designed for straightforward placement on general body landmarks, without requiring vertebral-level identification, making them more practical for occupational and field-based measurement [29]. Whether such systems can accurately resolve independent thoracolumbar and lumbosacral kinematics across all planes of motion during lifting has not been established.

Beyond sensor hardware, the software pipeline used to derive joint angles from IMU signals also influences the resulting kinematics. Different IMU analysis pipelines are available for estimating vertebral joint kinematics. In commercial systems, proprietary software can estimate thoracolumbar and lumbosacral kinematics as part of a full-body kinematic reconstruction. For example, Xsens has developed a 17-sensor full-body IMU system that uses proprietary sensor fusion algorithms and a custom biomechanical model with four trunk segments [29]. Alternatively, open source platforms such as OpenSense provide an IMU-based kinematics tool within the open-source musculoskeletal modeling platform OpenSim [30]. These tools offer a freely accessible alternative that is compatible with the same complex, validated thoracolumbar musculoskeletal models commonly used as a gold standard for estimating spine kinematics during lifting [13,14]. Importantly, OpenSense is compatible with Xsens sensor fusion-processed data, meaning the same IMU hardware can be used with both pipelines. In addition, while the MVN Analyze software allows flexible configurations and technically requires a minimum of eight sensors, trunk kinematics is estimated using only two sensors (i.e., one on the pelvis and one on the thorax). In contrast, OpenSense has the potential to directly estimate trunk motion using just these two sensors, making it highly practical for field-based measurements. Originally validated for lower-limb kinematics during walking [30], OpenSense has recently been applied to evaluate three-degrees-of-freedom (DOFs) trunk kinematics (i.e., relative angles between thorax and pelvis) during single plane range-of-motion tasks and simulated surgery tasks using four IMUs [11], but has not yet been evaluated using two IMUs for estimating six DOFs coordinate coupler constrained trunk kinematics (i.e., thoracolumbar and lumbosacral motion) during lifting movements. This leaves several important questions unaddressed. First, how valid are T12/L1 and L5/S1 joint angles produced by the Xsens MVN Analyze pipeline relative to those obtained from the validated OpenSim musculoskeletal lifting model using OMC gold standard data? Second, if differences exist between Xsens MVN Analyze and OMC, to what extent are they attributable to the underlying biomechanical model used by XSens, and does processing the same IMU sensor-fusion-derived data through OpenSense and the validated musculoskeletal model of OpenSim improve agreement? Third, how valid is OpenSense itself for estimating thoracolumbar and lumbosacral kinematics during lifting, and to what extent do the two IMUs pipelines produce meaningfully different joint kinematics? Answering these questions has direct practical relevance for researchers and practitioners who require accurate spinal kinematic measurement but may not have access to commercial software.

Therefore, the aim of the present study is to validate two IMU-based pipelines (i.e., Xsens MVN Analyze model and OpenSense combined with the validated OpenSim model, both using Xsens sensor fusion data as input) against the gold-standard OMC data processed through the validated OpenSim musculoskeletal model for quantifying thoracolumbar and lumbosacral kinematics across all planes of motion during symmetric and asymmetric lifting tasks. Specifically, we aim to (1) quantify the agreement between each IMU-based pipeline and OMC for T12/L1 and L5/S1 joint angles in the sagittal, frontal, and transverse planes and (2) determine whether meaningful kinematic differences exist between the Xsens MVN Analyze and OpenSense pipelines. Based on the demonstrated accuracy of both systems in related movement tasks [10,11], we hypothesize that both Xsens MVN Analyze and OpenSense would show acceptable agreement with OMC (errors within 5°) and that kinematic waveforms derived from the two IMU pipelines would not differ meaningfully from each other or the OMC pipeline.

## 2. Materials and Methods

### 2.1. Participants

A total of 9 healthy adults (6 male, 3 female, age = 22.2 ± 7.7 yrs., height = 178.1 ± 10.8 cm, weight = 73.9 ± 12.6 kg) volunteered to participate in this study. Participants provided informed consent, and the protocol was approved by MedStar Health Research Institute Institutional Review Board (IRB STUDY00009399).

### 2.2. Experimental Procedure

**Figure 1:**
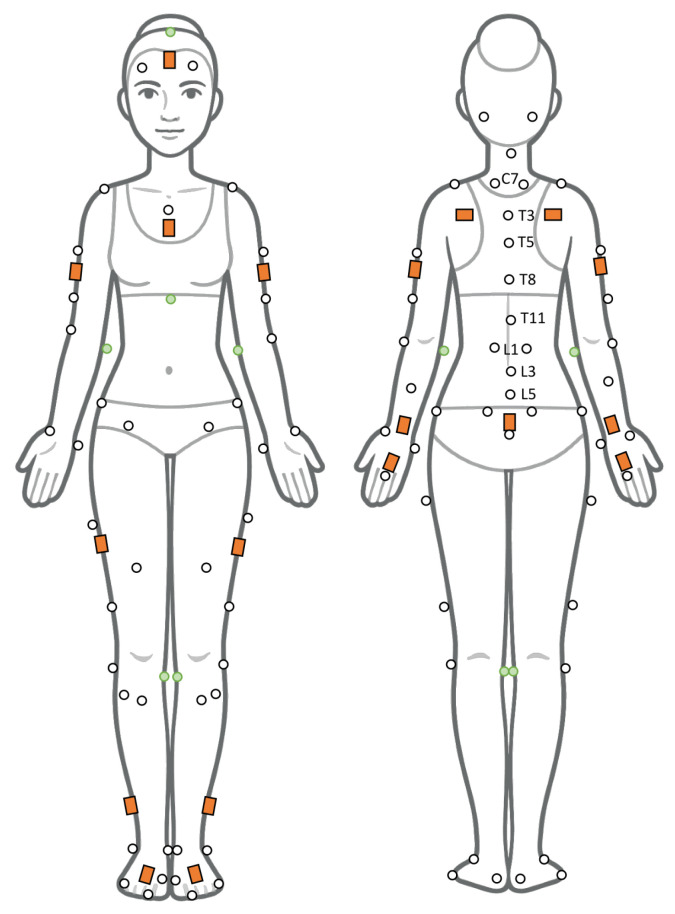
Retroreflective markers (white and green circles) and Xsens IMU sensors (orange squares) placement. Markers with green shading were removed after static calibration. Xsens IMU sensors were placed according to manufacturer instructions. Reflective markers were placed across head, C4, xiphoid process, sternoclavicular joint, sacrum, labeled spinal processes and bilaterally on acromion processes, medial and lateral elbow epicondyles, radial and ulnar styloid processes, third metacarpophalangeal joints, anterior and posterior superior iliac spines, iliac crest, medial and lateral femoral epicondyles, medial and lateral malleoli, calcaneus, 1st and 5th metatarsophalangeal joints, and hallux.

A total of 69 reflective markers and 17 IMU sensors were attached to the body as shown in Figure 1. IMUs were placed on the feet, legs, pelvis, trunk, arms, and head according to the manufacturer instructions. The Xsens calibration procedure was performed and then a standing n-pose static calibration trial was captured by all systems. This calibration procedure was performed only once at the beginning of the data collection. Participants executed lifting motions while movement was recorded using OMC (NEXUS software with 12 VERO infra-red cameras, VICON Motion Systems), and IMU sensors (MVN analyze Pro software version 2025.2 with 17 MVN Awinda sensors, Xsens Technologies N.A. Inc., Enschede, The Netherlands) at sampling frequencies of 100 Hz and 60 Hz, respectively. All instruments were hardware-synchronized, according to manufacturer recommendations. Participants lifted a box of 10 kg for 1 min using 4 lifting styles in a randomized block design order: (1) a stoop technique (knees extended), (2) a semi-squat technique (90° knee flexion and 45° trunk flexion), (3) a free technique (participant preference, no instruction), and (4) asymmetric lifting. For asymmetric lifts the box was lifted and placed approximately 60 degrees to the right and left sides of participants. Cadence was guided by a computer-generated metronome set to 12 beats per minute, with each lifting cycle performed 6 times during each one-minute trial. Each lifting cycle consisted of (i) reaching for the weight at the first beat, (ii) lifting it to waist level, (iii) pausing briefly, (iv) lowering and releasing the weight at the second beat, and (v) returning to the starting position.

### 2.3. Musculoskeletal Models

The OpenSim top–down, full-body model of the thoracolumbar spine used in this study has been validated for lifting tasks [14,15,16,17,31], and it consists of 78 rigid bodies and 165 DOFs. The thoracolumbar spine comprises 17 rigid bodies with 51 DOFs. Coordinate coupler constraints were applied to reduce the 51 DOFs of the thoracolumbar spine to 6 DOFs while computing kinematics [13], resulting in a thoracolumbar spine comprising 4 sections (Flexion/Extension (FE): T1–T9, T9–S1, Lateral Bending (LB): T1–L1, L1–S1, Axial Rotation (AR): T1–L1, L1–S1). The Xsens MVN Analyze Pro full-body model consists of 23 segments connected by 22 joints, with each segment tracked in six DOFs (three translational and three rotational) to capture full 3D kinematics [29]. The Xsens model thoracolumbar spine comprises 4 segments (T1–T8, T9–T12, L1–L3 and L4–L5).

### 2.4. Data Analysis

Custom scripts were used to run OpenSim’s Inverse Kinematics in MATLAB R2024b (The Mathworks Inc., Natick, MA, USA). For all systems, each spinal body segment was defined with the X-axis pointing forward, the Y-axis along the long axis of the segment, and the Z-axis defined by the right-hand rule.

OMC: OMC data were labeled in Vicon Nexus Software (2.16), and the resulting processed .c3d files were input to OpenSim 4.4 musculoskeletal modeling software [12] to compute the inverse kinematics. First, the marker data from the static calibration trial were used to scale female and male musculoskeletal models to each participant. Joint kinematics of the dynamic trials were then computed using the OpenSim Inverse Kinematics tool with each participant’s scaled musculoskeletal model using the ZXY rotation sequence. Resulting kinematic data were filtered with a dual-pass 4th-order Butterworth filter with a cutoff frequency of 6 Hz. This filter type and cutoff frequency were selected based on previous research utilizing similar marker sets, musculoskeletal models, and tasks [11,13,16].

Xsens: Anthropometrics for each subject were entered into the MVN Analyze software to estimate segment lengths. The Xsens MVN Analyze software, version 2025.2 automatically uses a proprietary Kalman Filter to process the IMU sensor data. Trial data were reprocessed with slow high-definition quality setting to optimize accuracy before exporting Xsens MVN Analyze computed joint angles. The IMU joint kinematics were exported from Xsens software using the ZXY rotation sequence option.

OpenSense: Sensor orientation quaternions were exported from Xsens MVN software for both the standing static calibration trial and dynamic lifting trials. Using the OpenSense IMU Placer Tool, the static calibration quaternions were used to orient and scale the OpenSim thoracolumbar spine model by aligning the virtual IMU locations and orientations on the model to those recorded during the static trial. The OpenSense IMU Inverse Kinematics Tool was then used to estimate joint kinematics for each lifting trial by minimizing the error between the orientations of the virtual IMUs on the calibrated model and the recorded IMU orientations.

For asymmetric lifting trials, kinematics from lifts performed in the left-to-right direction were mirrored across 50% of the lift cycle in the lateral and axial directions to match the coordinate system orientation of the right-to-left lifts. This allowed all six asymmetric lifts to be analyzed together when comparing kinematics across systems. Calculated L5/S1 and T12/L1 joint angles were extracted for OMC, OpenSense, and Xsens analyses. The velocities of the T3, T8, and L3 markers were used to identify the start and end of the lifting cycle based on transitions to and from near-zero velocity. An additional 0.5 s was added before and after each detected event to capture a brief period in which the participant was standing upright in a neutral position prior and following trunk movement. Data were time-normalized over 101 points to represent 0 to 100% of the lifting cycle. For each variable, the entire signal waveform was extracted as well as range of motion (ROM) values. and these values were averaged across the 6 trials for all 9 participants.

### 2.5. Statistical Analysis

To satisfy the independence assumptions underlying the statistical analyses, kinematic waveforms and the ROM values were averaged across the six repeated trials within each participant and lift type. Each statistical analysis was conducted on subject-level data (*n* = 9). Validity between systems was assessed across the entire kinematic waveform using root mean squared error (RMSE) and mean absolute error (MAE). To evaluate main effects across the kinematic waveforms, for each lift type and plane of motion, one-way repeated-measures statistical parametric mapping (SPM) with system (OMC, Xsens, and OpenSense) as the within-subject factor was applied (α=0.05) [32]. If significant main effects were detected, pairwise differences between systems were examined using post hoc SPM paired *t*-tests with Bonferroni correction (α=0.05/3=0.0167). One-dimensional SPM allows for time-series analysis of kinematic data by continuously sampling over time with Random Field Theory (RFT). RFT treats the normalized 0 to 100% waveform as a smooth curve to calculate a critical threshold while accounting for correlation between data points. Significance is detected when the scalar test statistic (i.e., t- or F-statistic) calculated at each time point crosses this critical threshold. Regions where the test statistic exceeds the critical threshold identify the specific percentage of the lift cycle during which significant differences occur. When there are significant differences between systems, post hoc SPM tests can be applied at the individual regions. This analysis was applied to spinal kinematic waveforms using the open-source spm1d package. Validity of ROM values was assessed using Bland–Altman analysis with Limits of Agreement (LoA) (α=0.05) and 95% Confidence Intervals (CIs) and Concordance Correlation Coefficients (CCCs) [33]. CCCs were interpreted as less than 0.5 as poor, moderate 0.5 to 0.75, good 0.75 to 0.9, and excellent greater than 0.9. For Bland–Altman analyses, proportional bias was assessed using linear regression of the differences against the means (β, p<0.05).

## 3. Results

### 3.1. Thoracolumbar

**Figure 2:**
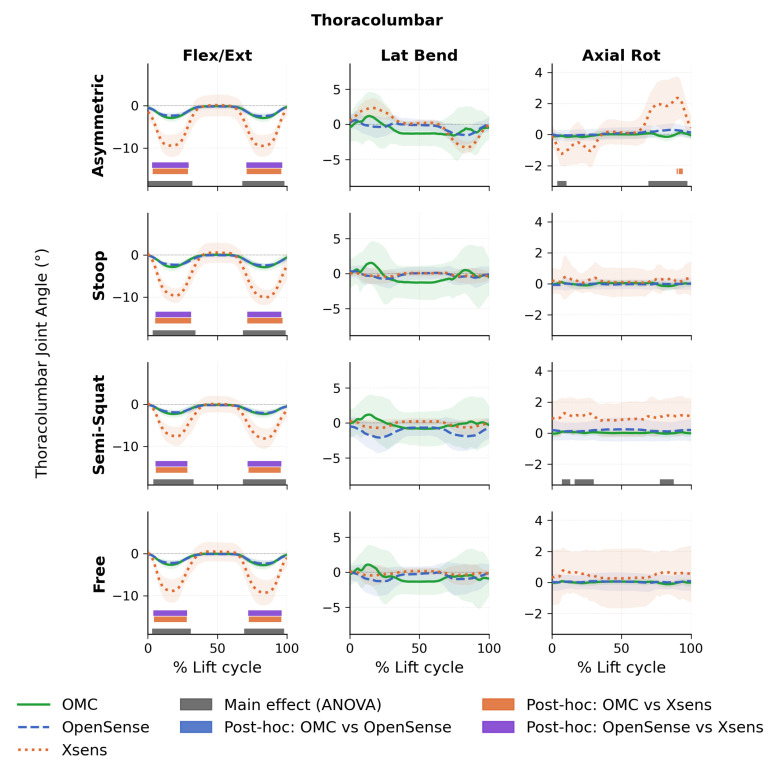
Thoracolumbar Mean joint angles for optical motion capture (OMC), OpenSense, and Xsens systems for all lifting tasks and dimensions. Shaded region indicates ±1 standard deviation. Main effects are shown in gray horizontal bars, and post hoc differences are shown in orange, blue, and purple horizontal bars.

Results of SPM analysis for the thoracolumbar joint are shown in Figure 2. Significant main effects between systems were observed for all lift types in the flexion-extension direction. In the axial rotation direction, significant main effects were present during asymmetric and semi-squat lifts only. No significant main effects were found in the lateral bending direction for any lift type, and no significant main effects were found for stoop or free lifts in the axial rotation direction. Post hoc tests revealed differences only between Xsens and OMC and between Xsens and OpenSense, whereas no differences were observed between OpenSense and OMC for any direction or lift type. More in detail, for flexion-extension, differences between OMC and Xsens were observed during semi-squat (5.7–28.5% and 72.2–95.4% of the movement cycle, p<0.001), stoop (5.2–31.1% and 71.5–96.7%, p<0.001), asymmetric (3.5–28.9% and 71.0–95.9%, p<0.001), and free lifts (4.4–28.0% and 72.5–96.0%, p<0.001). Similarly, differences between OpenSense and Xsens in flexion-extension were also present across all lift types: semi-squat (5.4–28.5% and 71.7–96.0%, p<0.001), stoop (5.5–31.0% and 71.5–96.0%, p<0.001), asymmetric (3.1–29.3% and 70.7–96.6%, p<0.001), and free lifts (3.9–28.3% and 72.1–96.1%, p<0.001). In the axial rotation direction, post hoc tests showed differences between OMC and Xsens during asymmetric lifts (89.6–90.6%, p=0.016; 91.1–94.2%, p=0.010). No significant post hoc differences were identified between OpenSense and Xsens or between OMC and OpenSense in the axial rotation direction.

Table 1 lists the RMSE and MAE between each system pair for T12/L1 joint angles across all lift types. OMC vs. Xsens errors were largest in the flexion-extension plane, with RMSE ranging from 3.7 to 4.7° across all lift types, while RMSE in the lateral bending and axial rotation planes ranged from 0.9 to 3.2°. OMC vs. OpenSense showed generally excellent agreement across all planes, with RMSE ranging from 0.4 to 3.2°. For OpenSense vs. Xsens, errors were again largest in the flexion-extension plane (RMSE: 3.7 to 4.8°), with comparatively smaller errors in the lateral bending and axial rotation planes (RMSE: 0.9 to 1.9°).

**Figure 3:**
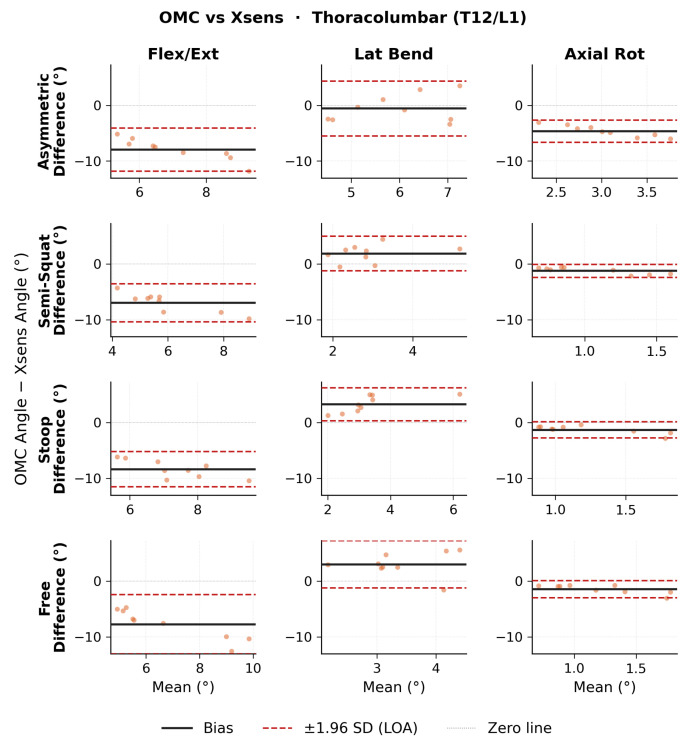
Bland–Altman plots of thoracolumbar range of motion (ROM) joint angles between optical motion capture (OMC) and Xsens for each lifting task in each plane of movement.

**Figure 4:**
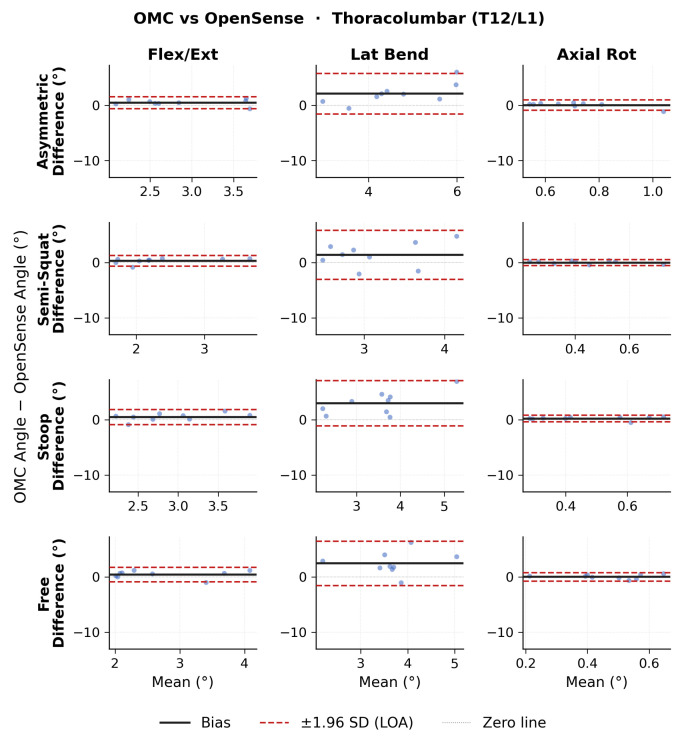
Bland–Altman plots of thoracolumbar range of motion (ROM) joint angles between optical motion capture (OMC) and OpenSense for each lifting task in each plane of movement.

**Figure 5:**
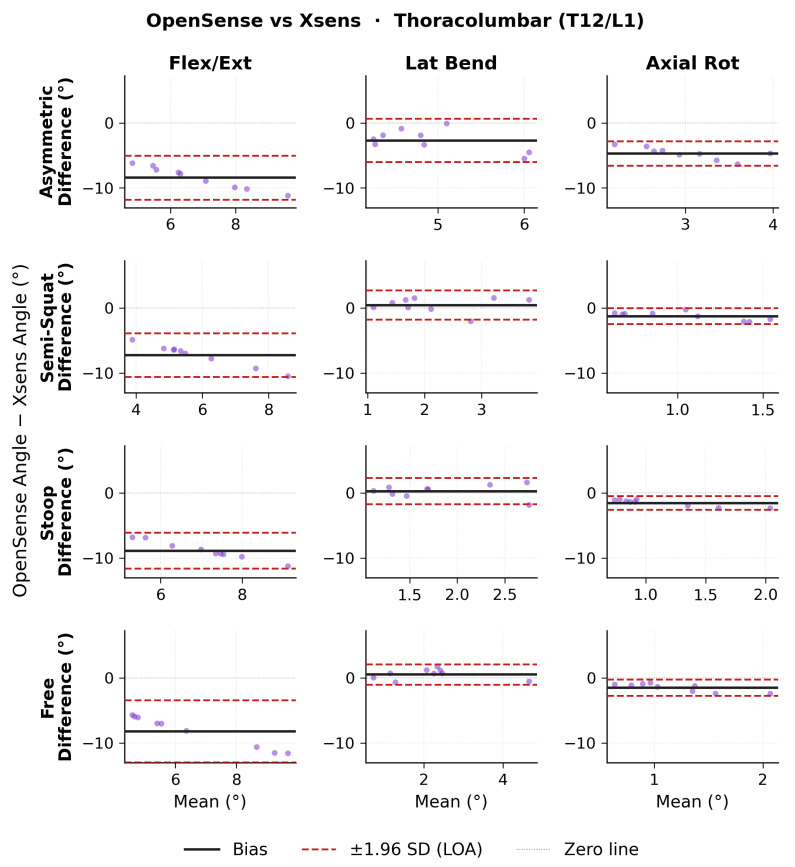
Bland–Altman plots of thoracolumbar range of motion (ROM) joint angles between OpenSense and Xsens for each lifting task in each plane of movement.

Bland–Altman results for ROM thoracolumbar angles are shown in Figure 3 for OMC vs. Xsens, Figure 4 for OMC vs. OpenSense, and Figure 5 for OpenSense vs. Xsens. Thoracolumbar ROM angle agreement followed similar patterns to lumbosacral ROM angles. Numerical results of bias, 95% CI, and upper and lower LoA are presented in Table 2. OMC vs. Xsens showed consistent negative flexion-extension biases across all lift types (−6.9 to $-8.3^{\circ }$; LoA: ±3.2 to $\pm 5.3^{\circ }$), with Xsens overestimating T12/L1 flexion. OMC vs. OpenSense biases were negligible in flexion-extension (0.3 to $0.5^{\circ }$; LoA: ±1.0 to $\pm 1.4^{\circ }$) but showed wider LoA in the lateral bending plane. OpenSense vs. Xsens showed consistent negative flexion-extension biases across all lift types (−7.2 to $-8.8^{\circ }$; LoA: ±2.8 to $\pm 4.8^{\circ }$), with Xsens overestimating T12/L1 flexion relative to OpenSense. In the lateral bending and axial rotation planes, biases were small but LoA were wide in the lateral bending plane.

Thoracolumbar CCC values for ROM are presented in Table 3. OMC vs. Xsens showed poor agreement across all planes (CCC < 0.1). OMC vs. OpenSense demonstrated poor to moderate agreement in flexion-extension (CCC: 0.34 to 0.70), while agreement in lateral bending and axial rotation was poor and inconsistent across lift types, with several negative values. OpenSense vs. Xsens similarly showed poor agreement in all planes (CCC < 0.20) besides lateral bending for free lifts (CCC = 0.65).

### 3.2. Lumbosacral

**Figure 6:**
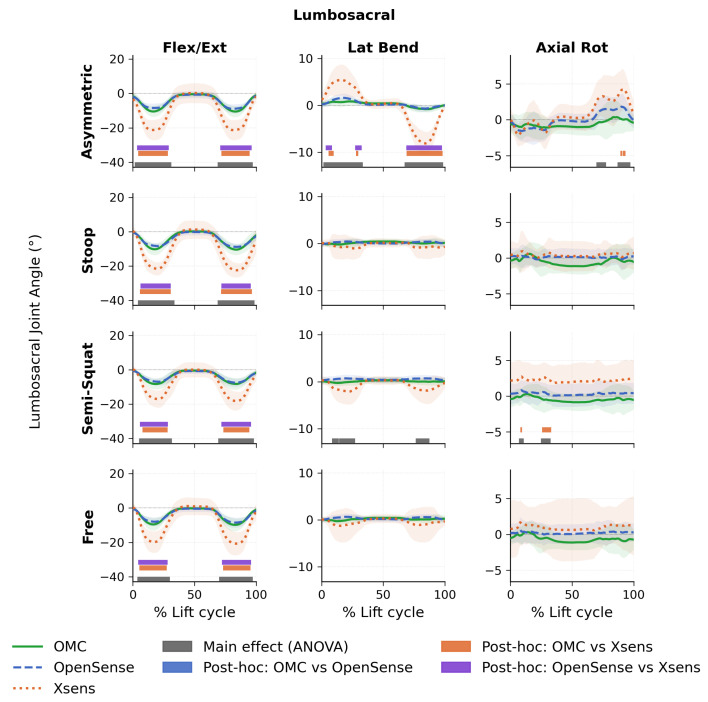
Lumbosacral Mean joint angles for optical motion capture (OMC), OpenSense, and Xsens systems for all lifting tasks and dimensions. Shaded region indicates ±1 standard deviation. Main effects are shown in gray horizontal bars, and post hoc differences are shown in orange, blue, and purple horizontal bars.

Results of SPM analysis for the lumbosacral joint are shown in Figure 6. Significant main effects between systems were seen for all types of lift in the flexion-extension direction. In the lateral bending and axial directions, significant main effects were present during asymmetric and semi squat lifts, while no significant main effects were found for stoop or free lifts in the lateral bending or axial rotation directions. Post hoc tests revealed that in the flexion-extension direction, differences were significant only between Xsens and OMC and between Xsens and OpenSense, while no differences were observed between OpenSense and OMC for any lift type. More in detail, differences in flexion-extension between OMC and Xsens were observed during semi-squat (7.7–28.3% and 73.5–94.6% of the movement cycle, p<0.001), stoop (5.6–30.8% and 71.6–96.7%, p<0.001), asymmetric (4.4–28.4% and 71.7–94.8%, p<0.001), and free lifts (5.2–27.4% and 73.3–95.5%, p<0.001). Similarly, differences between OpenSense and Xsens in flexion-extension were also present across all lift types: semi-squat (5.8–28.4% and 71.9–95.8%, p<0.001), stoop (6.1–30.7% and 71.7–95.7%, p<0.001), asymmetric (3.3–29.2% and 70.9–96.3%, p<0.001), and free lifts (4.3–28.1% and 72.3–95.8%, p<0.001). In the lateral bending direction, post hoc tests revealed differences between OMC and Xsens (5.6–9.8%, p=0.004; 27.8–29.7%, p=0.012; and 69.0–98.1%, p<0.001), and between OpenSense and Xsens during asymmetric lifts (3.3–8.4%, p=0.002; 27.1–32.6%, p=0.002; and 68.6–97.6%, p<0.001).

In the axial rotation direction, post hoc tests revealed differences between OMC and Xsens during asymmetric lifts (91.1–93.4%, p=0.014) and during semi-squat lifts (8.0–9.4%, p=0.015; 25.7–33.2%, p=0.002).

Table 4 lists the RMSE and MAE between each system pair for L5/S1 joint angles across all lift types. OMC vs. Xsens errors were largest in the flexion-extension plane, with RMSE ranging from 6.5 to 8.5° across all lift types, while RMSE in the lateral bending and axial rotation planes ranged from 1.4 to 4.0°. OMC vs. OpenSense showed generally excellent agreement across all planes, with RMSE ranging from 0.8 to 2.3°. For OpenSense vs. Xsens, errors were again largest in the flexion-extension plane (RMSE: 6.7 to 8.8°), with comparatively smaller errors in the lateral bending and axial rotation planes (RMSE: 1.2 to 3.9°).

**Figure 7:**
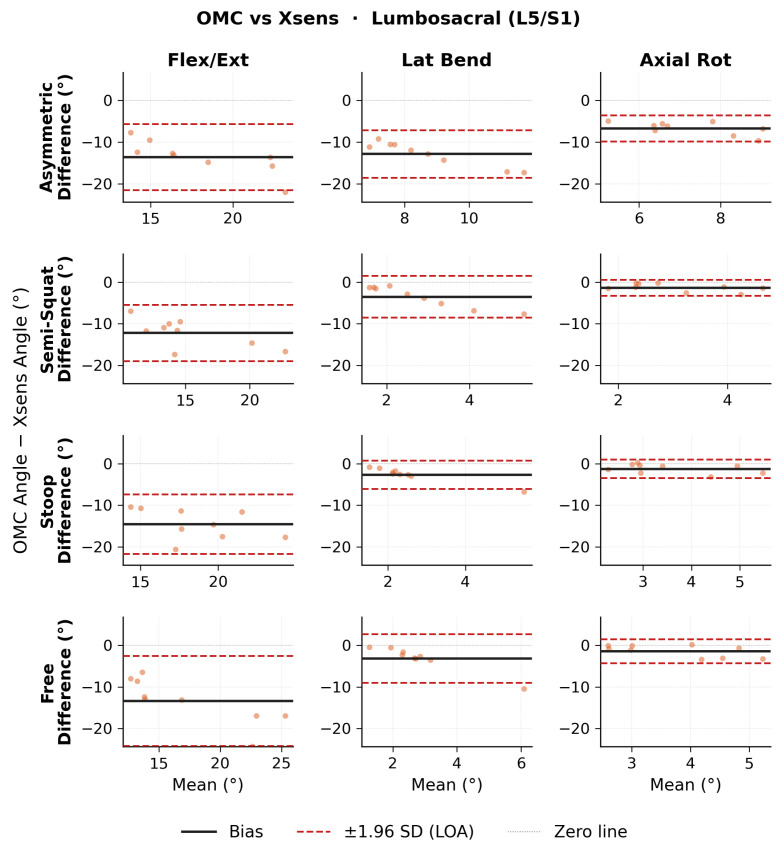
Bland–Altman plots of lumbosacral range of motion (ROM) joint angles between optical motion capture (OMC) and Xsens for each lifting task in each plane of movement.

**Figure 8:**
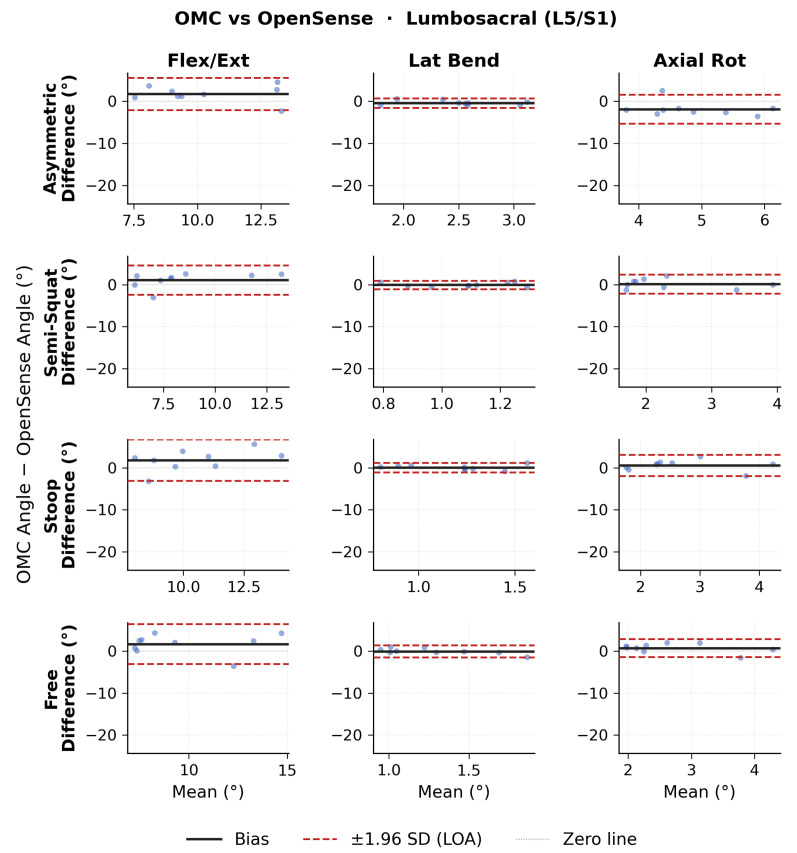
Bland–Altman plots of lumbosacral range of motion (ROM) joint angles between optical motion capture (OMC) and OpenSense for each lifting task in each plane of movement.

**Figure 9:**
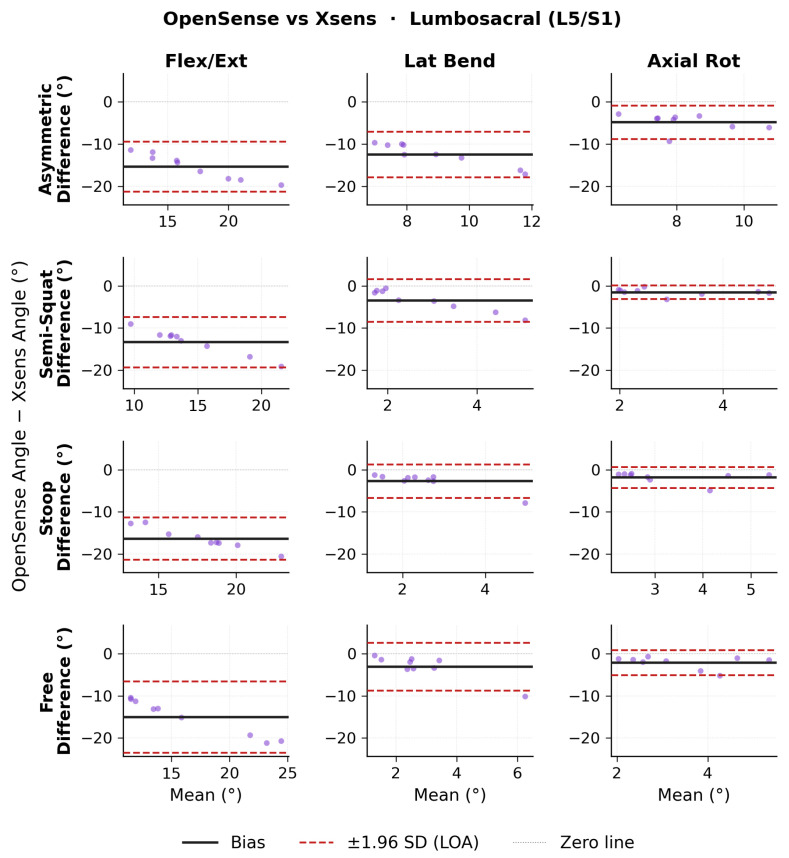
Bland–Altman plots of lumbosacral range of motion (ROM) joint angles between OpenSense and Xsens for each lifting task in each plane of movement.

Bland–Altman results for ROM lumbosacral angles are shown in Figure 7 for OMC vs. Xsens, Figure 8 for OMC vs. OpenSense, and Figure 9 for OpenSense vs. Xsens. Numerical results of bias, 95% CI, and upper and lower LoA are presented in Table 5. OMC vs. Xsens showed large systematic biases in flexion-extension across all lift types (−12.2 to $-14.5^{\circ }$; LoA: ±6.7 to $\pm 10.8^{\circ }$), with Xsens overestimating L5/S1 flexion. OMC vs. OpenSense biases were smaller (1.1 to $1.8^{\circ }$; LoA: ±3.5 to $\pm 4.9^{\circ }$). OpenSense vs. Xsens showed similarly large negative flexion-extension biases across all lift types (−13.3 to $-16.3^{\circ }$; LoA: ±5.0 to $\pm 8.5^{\circ }$), with Xsens overestimating L5/S1 flexion relative to OpenSense. In the lateral bending and axial rotation planes, biases were relatively small across all comparisons but LoA were wide.

Lumbosacral CCC values for ROM are presented in Table 6. Agreement between OMC and Xsens was poor across all planes and lift types (CCC < 0.15). Agreement between OMC and OpenSense was poor to moderate in the flexion-extension plane across all lift types (CCC: 0.34–0.70). In the lateral bending and axial rotation planes, agreement was poor across all lift types (CCC < 0.34), with several negative values indicating systematic disagreement in direction as well as magnitude. Agreement between OpenSense and Xsens was similarly poor across all planes (CCC < 0.45).

## 4. Discussion

The aim of this study was to validate two IMU based pipelines, Xsens MVN Analyze and OpenSense, against gold-standard OMC measurements processed through a validated OpenSim musculoskeletal model for quantifying T12/L1 and L5/S1 joint kinematics across all planes of motion during symmetric and asymmetric lifting tasks. A secondary aim was to determine whether meaningful kinematic differences existed between the two IMU pipelines. Based on previous research examining single segment trunk angles with Xsens IMU during lifting, we hypothesized there would be excellent agreement, errors within $5^{\circ }$, and no significant differences between joint angles.

This hypothesis was not supported for all joint angles and planes of motion. Xsens MVN Analyze showed large differences in the flexion-extension plane at both spinal levels across all lift types as compared to the gold-standard OMC measurements. At the lumbosacral joint, Bland–Altman biases of ROM ranged from −12.2 to $-14.5^{\circ }$ with LoA of approximately ±6.7 to $\pm 10.8^{\circ }$, indicating that Xsens greatly overestimated L5/S1 flexion. At the thoracolumbar joint, biases were somewhat smaller but still exceeded the acceptable $5^{\circ }$ threshold (−6.9 to $-8.3^{\circ }$; LoA: ±3.2 to $\pm 5.3^{\circ }$). CCC values between Xsens and OMC were poor across all planes of motion for both lumbosacral and thoracolumbar ROM (CCC < 0.15), consistent with the large systematic biases observed in Bland–Altman analysis, as CCC is highly sensitive to systematic bias. The Bland–Altman results highlight potential systematic error when utilizing Xsens MVN model and software with IMU sensors to measure thoracolumbar and lumbosacral joint angles. For the OMC vs. Xsens and OpenSense vs. Xsens ROM comparisons there is a proportional bias in all planes of motion. This bias indicates that the magnitude of agreement is somewhat dependent on the magnitude of the measured angle. Proportional bias was statistically significant for the majority of flexion-extension and lateral bending comparisons involving Xsens, with slopes ranging from approximately −0.5 to $-2.2^{\circ }$ at the lumbosacral joint and −1.0 to $-1.7^{\circ }$ at the thoracolumbar joint. These findings indicate that Xsens overestimation increased progressively with joint angle ROM magnitude. One potential explanation for this proportional bias is sensor calibration error, which may become more pronounced at larger joint angles. However, unlike comparisons involving MVN Analyze, there was no evidence of proportional bias across most lift types and planes of motion between OpenSense and OMC, suggesting that the proportional bias observed in MVN Analyze-derived joint angles is more likely attributable to differences in modeling approaches. SPM analysis of the entire spinal joint angle waveforms confirmed these flexion-extension differences were statistically significant during the lifting and lowering phases of all lifting cycles, when peak angles occur. Agreement in lateral bending and axial rotation was comparatively better, with lower biases and smaller LoA, and significant post hoc differences limited to lumbosacral lateral bending during asymmetric lifting and axial rotation during asymmetric and semi-squat lifts.

Although the goal is not to examine differences in IMU pipeline validity across lift types, some patterns suggest potentially lower validity during asymmetric lifting with Xsens MVN sensors and software compared to other systems. While waveform differences and errors were predominant in the flexion-extension plane across all lift types and joints, errors appeared across multiple planes during asymmetric lifting. Specifically, axial rotation differences between OMC and Xsens were observed at both joints near the end of the lift, and lumbosacral lateral bending errors between Xsens and both OMC and OpenSense were present during the lifting and lowering phases. Lumbosacral bias, RMSE, and MAE during lateral bending also appeared relatively larger for asymmetric lifting, and thoracolumbar axial rotation bias appeared greater, though thoracolumbar errors did not differ notably across lift types. This likely occurs because asymmetric lifting introduces movement outside the sagittal plane, including greater axial rotation and lateral bending, which may challenge the Xsens MVN sensor fusion and modeling assumptions, leading to reduced validity compared to more constrained, primarily sagittal-plane lift types. Together, these patterns suggest that asymmetric lifting may present additional validity challenges for the Xsens MVN system, especially in movements occurring outside the sagittal plane, and should be interpreted with greater caution for this lift type.

To examine whether these differences in lumbosacral and thoracolumbar joint angles could be partially attributed to model differences between the OpenSim musculoskeletal model and the Xsens MVN Analyze biomechanical model, Xsens sensor fusion IMU quaternions were input into OpenSense. OpenSense demonstrated excellent agreement with OMC for both T12/L1 and L5/S1 joint angles across all lifting tasks and planes of motion, with RMSE values consistently below $3.2^{\circ }$ and Bland–Altman biases that were negligible in magnitude. Bland–Altman biases ranged from 1.1 to $1.8^{\circ }$ for L5/S1 and 0.3 to $0.5^{\circ }$ for T12/L1 flexion-extension ROM, with somewhat larger biases in the lateral bending plane, particularly at the lumbosacral joint. These small errors and biases are well within the clinically acceptable threshold of $5^{\circ }$. However, CCC values between OMC and OpenSense were moderate in the flexion-extension plane (CCC > 0.53) across most lift types, with the exception of stoop lifts which showed poor agreement (CCC = 0.33). Agreement in the lateral bending and axial rotation planes was generally poor across all lift types. The moderate to poor CCC values in flexion-extension, despite small Bland–Altman biases and low RMSE values, likely reflect limited between-participant variability in ROM within this relatively small, homogeneous sample of young healthy adults. CCC is sensitive to the range of values in the dataset as well as bias, meaning that a small, homogeneous sample can produce moderate or poor CCC values even when two systems are producing clinically similar estimates. The poor CCC values in the lateral bending and axial rotation directions indicate that although absolute differences between systems may be small, the correlation between signals may also be low. In these low-magnitude planes, even small random errors represent a large proportion of the signal, which can reduce CCC. Therefore, low bias alone is insufficient to conclude good agreement in these planes, and results in the lateral bending and axial rotation directions should be interpreted with caution. Additionally, when comparing both IMU pipelines, the large errors, biases, LoA, and CCC observed between Xsens MVN Analyze and OpenSense further suggest that the poor validity of Xsens MVN Analyze is driven primarily by differences in the underlying biomechanical model rather than the IMU sensor technology itself.

A main consideration when interpreting these results is the differences in the biomechanical models used by Xsens and OpenSim. The previously developed top-down thoracolumbar full-body OpenSim model was used for both OMC and OpenSense data, while Xsens software uses a built-in proprietary model. To account for differences in joint angle offsets between the Xsens MVN Analyze model and the OpenSim model, joint angles during each trial were expressed relative to the standing neutral position. This approach removes any offset introduced by segment definitions and enables direct comparison of joint motion during lifting. This normalization may explain why SPM waveform differences were not seen during the lifting cycle where the participant is standing upright and still while holding the box.

Alongside differences in model structure, the methods used to calculate joint angles may differ between IMU pipelines. Based on best practices, coordinate coupler constraints were applied to the OpenSim model to reduce the number of degrees of thoracolumbar freedom from 51 to 6, which has been shown to produce physiological feasible spine kinematics during lifting [13]. The Xsens modeled trunk contains a pelvis, T1–T8, T9–T12, L1–L3, L4–L5, segments all modeled with three DOFs, but only pelvis and T1–T8 are defined based on IMU sensors [29]. The kinematics of other segments, L4–L5, L1–L3, and T9–T12 are estimated based on the biomechanical model and defined according to ISB recommendations [34]. Without kinematic constraints, thoracolumbar range of motion can exceed physiological values [13], and the unconstrained Xsens model may partially account for the overestimated joint angles recorded by Xsens during lifting. When comparing OpenSense to Xsens, flexion/extension RMSE ranged from $3.7^{\circ }$ to $8.8^{\circ }$ across the L5/S1 and T12/L1 joints. This is consistent with previous work demonstrating that differences in trunk angles during lifting are largely attributable to model differences, with mean RMSE ranging from 3.4° to 6.1° [10]. These values are comparable to the T12/L1 flexion-extension RMSE observed between OpenSense and Xsens in the present study. However, the corresponding L5/S1 errors were notably larger, suggesting that model-related differences may exert a greater influence on lumbosacral than thoracolumbar kinematics.

Ultimately, the comparison of OpenSense to Xsens confirmed that the two pipelines produce meaningfully different joint kinematics despite receiving identical IMU sensor fusion orientations as input. At the lumbosacral joint, Xsens consistently estimated greater flexion ROM RMSE than OpenSense by 6.7 to $8.8^{\circ }$ across all lift types, and by 3.7 to $4.8^{\circ }$ at the thoracolumbar joint. SPM analysis identified statistically significant flexion-extension differences across all lift types and at both joints, occurring during the same lifting and lowering cycle phases as the OMC vs. Xsens differences. CCC values between OpenSense and Xsens were generally poor across all planes of motion for both joints (CCC range: −0.40 to 0.69), with flexion-extension values consistently near zero (CCC < 0.10), further corroborating the large systematic differences observed in RMSE and Bland–Altman analyses. Together, these findings suggest that the Xsens biomechanical model, despite representing the trunk as a multi-segment spine, may not be sufficiently constrained to accurately capture thoracolumbar and lumbosacral joint kinematics during lifting. It should be noted, however, that these interpretations assume the OpenSim musculoskeletal model and its associated pipelines represent the gold standard for spinal kinematic estimation.

Previous lifting research with the Xsens and OpenSense pipeline is limited, but a prior study utilized OpenSense to examine trunk kinematics during a sagittal-plane motion task and a simulated surgery task. Xsens MTw IMUs were placed on the back of the head, sternum, T5, and above T10. Similar to the current study, but with a single rigid segment trunk, kinematics were estimated with ROM differences and RMSE less than 5° [11]. This previous study used the same full-body thoracolumbar OpenSim model applied in the current study, but this previous study locked L5 to T1 joints.

Although to the knowledge of the authors, this is the first study to use Xsens MVN Analyze sensor fusion data and complex musculoskeletal models to estimate spinal L5/S1 and T12/L1 joint angles during lifting, the results of this study can be compared to research where sensor placement was ultrasound guided or clinician palpated to measure global or spinal level joint angles. A previous study estimated thoracic and lumbar kinematics during leaning, bending, and twisting trunk movements using Xsens Dot IMUs placed over the C7, T12, and S1 segments, and reported ROM errors within 3° [27]. Similarly, a unique configuration of two IMUs was used, connected by a flexible rod with a potentiometer, positioned over S1 and T1, and reported RMSE within 3° [35]. A study evaluating the concurrent validity of the DorsaVi Version 6 system for peak flexion analysis of the global sacral and thoracic region angles reported ROM RMSE within 2° during lifting [26]. Overall, the errors shown in these previous studies examining spinal joint level kinematics across various tasks and with differing IMUs or pipelines reflect the findings of the current study.

However, when modeling the entire trunk as a single rigid segment and comparing joint angles to proprietary software calculated values, larger errors have been reported for lifting tasks. Using a full-body 17-sensor Xsens MTw IMU setup with proprietary Xsens MVN Analyze software, lifting trunk angle errors ranged were within 10° [7]. A similar full-body Xsens MVN Awinda setup yielded trunk angle errors within 16.5° [8]. Using OMC and Noraxon IMUs to quantify trunk angles with respect to the vertical during asymmetric squat-pivot and stoop-twist lifting tasks, errors were within $9^{\circ }$ [9]. In this study, when comparing proprietary Xsens software with gold standard OMC joint angles, there were comparable, larger joint angles. OMC vs. Xsens L5/S1 RMSE was within $8.5^{\circ }$, and T12/L1 RMSE was within $4.7^{\circ }$. Overall, these results suggest caution when using Xsens MVN Analyze software to interpret trunk or spinal joint angles.

One limitation of this study is that we did not examine a condition where the biomechanical model for OMC matches the biomechanical model of Xsens MVN Analyze. However, previous research has examined the Xsens model component in isolation from technological error and reported single segment trunk error during lifting [10]. This previous study found that the differences in single segment trunk angles were mostly due to model differences rather than IMU sensor technology, which aligns with our research findings. This current study focused on examining differences at the spinal joint level, but future work should examine a similar IMU-driven biomechanical modeling approach at each joint defined in the Xsens MVN model. This line of research would help isolate the relative contributions of sensor fusion, biomechanical model structure, and inverse kinematics assumptions to the observed differences.

An additional consideration between measurement systems was the choice of marker placement and IMU sensor location and the resulting soft tissue artifacts. Soft tissue artifact occurs when skin-mounted markers or sensors move relative to the underlying bone, and it has been established as a main source of measurement error [36]. In the lumbar region specifically, soft tissue artifact has been shown to increase with flexion angle [37]. Because IMU sensors and reflective markers are placed on different anatomical landmarks, the magnitude and direction of soft tissue artifact may differ between systems, and these differences may contribute to discrepancies in the kinematics derived from each. Importantly, soft tissue artifact errors in the lumbar region have been shown to be relatively lower in the mediolateral direction [37], suggesting that frontal plane kinematics, particularly lateral bending, are likely least affected by this source of error. Conversely, sagittal plane kinematics, capturing the primary flexion extension motion during lifting, are likely most susceptible to skin tissue artifact error, and discrepancies in this plane should be interpreted with this limitation in mind. Finally, it should be noted that optical motion capture specifically for spinal kinematics may not be a true gold standard as there are numerous markers that span the trunk highly susceptible to skin motion artifacts. Therefore, observed differences between systems may reflect limitations in both approaches rather than just error in the IMU sensor measurements.

Another limitation concerns the fact that the sensor fusion algorithm applied to the Xsens IMU data was also used for the OpenSense pipeline. As a result, the results of the sensor fusion step preceding the OpenSense analysis may differ if an independent, open-source sensor fusion algorithm were used instead. When data were recorded using Xsens MVN software, the proprietary Xsens Kalman Filter [29] had already been applied prior to export. This algorithm integrates accelerometer, gyroscope, and magnetometer data within the Xsens biomechanical model framework to estimate segment orientations, and because it is not openly available, it cannot be replaced or reproduced within the same data collection pipeline. Notably, the original research validated the OpenSense pipeline, compared the Xsens Kalman Filter against an open-source complementary filter, and found that the open-source filter outperformed the proprietary algorithm in reducing orientation drift over longer duration trials [30]. However, given that the lifting trials in the present study were short in duration, drift accumulation is unlikely to have been a meaningful source of error, and the influence of filter choice may be less consequential. Nevertheless, the effect of different sensor fusion algorithms on downstream spinal kinematic outputs in musculoskeletal modeling pipelines remains an open question and should be systematically examined in future research. Additionally, the resulting OMC data were filtered using a fourth-order dual-pass Butterworth Filter to reflect typical filtering practices in biomechanics research, while the Kalman Filter was applied to IMU data.

The lifting task was also performed under controlled conditions with a fixed speed, a set weight of 10 kg, and a structured format across four lift types, which may limit the generalizability of the findings to more variable real-world lifting scenarios. However, this level of standardization is advantageous for system validation, as it reduces behavioral variability and enables more direct attribution of differences between OMC, Xsens MVN, and OpenSense to the measurement systems themselves, thereby strengthening internal validity and supporting a fair comparison across systems. Future research should incorporate greater variation in speed, weight, and lifting style to better reflect the demands of real occupational tasks across a working day. Additionally, individuals only lifted for one minute per lift type, totaling six lifts, which does not capture the effects of fatigue or changes in attentional demand that occur over the course of a typical working day in an occupational setting. Future research should therefore examine the robustness of these IMU pipelines under conditions of prolonged activity and fatigue, where movement variability and compensatory strategies may influence kinematic estimates.

## 5. Conclusions

In conclusion, the biomechanical model used in the Xsens MVN Analyze pipeline consistently overestimated lumbosacral and thoracolumbar flexion-extension across all symmetric and asymmetric lift types examined, with biases exceeding clinically meaningful thresholds when compared against an OpenSim biomechanical model validated for lifting movements driven by gold-standard OMC data. In contrast, the OpenSense pipeline, when provided with the same full-body 17-sensor Xsens IMU quaternions and implemented within the same OpenSim biomechanical model used with the OMC data, demonstrated excellent agreement with gold standard OMC-derived T12/L1 and L5/S1 joint angles across all lifting conditions and planes of motion, with overall moderate CCC in flexion-extension and poor CCC in lateral bending and axial rotation planes, consistent with the limited between-participant ROM variability in this sample. These findings suggest that the observed discrepancies are primarily attributable to differences in biomechanical model implementation rather than limitations of the Xsens IMU sensors themselves or their underlying sensor fusion algorithms, which appear capable of providing accurate motion measurements when integrated with an appropriate modeling framework. While RMSE and Bland–Altman biases in the lateral bending and axial rotation planes were small, CCC values were generally poor, indicating that the two systems do not consistently track the same pattern of variation across participants in these planes. Therefore, IMU-derived lateral bending and axial rotation kinematics should be interpreted with caution. Moreover, the results suggest that using only two IMUs at the chest and on the pelvis can accurately estimate thoracolumbar and lumbosacral joint angles when the OpenSim spine in model is implemented with six DOF coordinate coupler constraints. Future work should examine the ability to use two IMUs at the chest and pelvis with custom sensor fusion algorithms in combination with OpenSense to further simplify sensor setup. In addition, future work should analyze whether adding additional IMUs along the spine could further increase accuracy while enabling spine models with a greater number of DOFs.

## Figures and Tables

**Table 1:** Thoracolumbar root mean squared error (RMSE) and mean absolute error (MAE) between systems across the entire lifting cycle. Values are mean ± SD and in degrees.

Lift	Plane	OMC vs. XSens		OMC vs. OpenSense		OpenSense vs. XSens	
		RMSE	MAE	RMSE	MAE	RMSE	MAE
Asym.	FE	4.5±1.4	3.7±1.3	0.5±0.3	0.4±0.2	4.6±1.2	3.8±1.0
	LB	3.2±1.2	2.7±0.9	2.8±1.6	2.4±1.5	1.9±0.6	1.5±0.5
	AR	1.5±0.4	1.3±0.4	0.5±0.2	0.5±0.2	1.6±0.4	1.3±0.4
Free	FE	4.2±1.3	3.5±1.1	0.5±0.2	0.4±0.2	4.2±1.1	3.5±0.9
	LB	3.1±1.4	2.7±1.1	3.2±1.8	2.8±1.8	1.4±0.7	1.3±0.7
	AR	1.3±1.2	1.2±1.2	0.6±0.2	0.5±0.2	1.2±0.9	1.1±1.0
Squat	FE	3.7±1.1	3.0±1.0	0.4±0.2	0.4±0.2	3.7±1.0	3.1±0.9
	LB	2.8±1.8	2.5±1.6	3.2±2.6	2.9±2.5	1.6±1.2	1.5±1.3
	AR	1.3±0.7	1.2±0.8	0.6±0.4	0.5±0.4	1.2±0.6	1.2±0.6
Stoop	FE	4.7±1.0	4.0±0.9	0.6±0.3	0.5±0.2	4.8±0.8	4.0±0.7
	LB	3.1±1.7	2.7±1.3	3.0±1.4	2.6±1.2	1.2±0.6	1.1±0.5
	AR	0.9±0.4	0.8±0.3	0.4±0.2	0.4±0.2	0.9±0.5	0.8±0.5

FE = flexion extension; LB = lateral bending; AR = axial rotation; Asym. = asymmetric.

**Table 2:** Bland–Altman Agreement: Range of Motion (ROM) of Thoracolumbar (T12/L1) joint angles (degrees) in each plane of motion, with 95% Confidence Interval (CI), Limits of Agreement (LoA), and proportional bias slope (β). Bold β indicates significance at α=0.05.

Lift	Plane	OMC vs. Xsens			OMC vs. OpenSense			OpenSense vs. Xsens		
		Bias [95% CI]	LoA	β	Bias [95% CI]	LoA	β	Bias [95% CI]	LoA	β
Asym.	FE	−7.9 [−9.4, −6.4]	±3.9	**−1.25**	0.5 [0.1, 0.9]	±1.1	−0.07	−8.4 [−9.7, −7.1]	±3.4	** −1.10 **
	LB	−0.6 [−2.5, 1.4]	±4.9	0.71	2.1 [0.7, 3.6]	±3.7	1.33	−2.7 [−4.0, −1.4]	±3.3	−1.44
	AR	−4.6 [−5.4, −3.8]	±2.0	** −2.06 **	0.1 [−0.3, 0.4]	±0.9	** −2.43 **	−4.7 [−5.4, −4.0]	±1.9	** −1.28 **
Semi-Squat	FE	−6.9 [−8.3, −5.6]	±3.4	** −1.04 **	0.3 [−0.1, 0.7]	±1.0	0.34	−7.2 [−8.6, −5.9]	±3.4	** −1.18 **
	LB	1.9 [0.7, 3.1]	±3.1	0.49	1.4 [−0.3, 3.1]	±4.4	1.26	0.5 [−0.4, 1.4]	±2.3	0.07
	AR	−1.2 [−1.7, −0.8]	±1.2	** −1.50 **	0.0 [−0.2, 0.2]	±0.6	−0.55	−1.2 [−1.7, −0.8]	±1.2	** −1.36 **
Stoop	FE	−8.3 [−9.6, −7.1]	±3.2	** −1.01 **	0.5 [−0.0, 1.1]	±1.4	0.63	−8.8 [−9.9, −7.8]	±2.8	** −1.18 **
	LB	3.3 [2.1, 4.5]	±3.0	0.92	3.0 [1.4, 4.6]	±4.1	1.53	0.3 [−0.5, 1.1]	±2.0	−0.06
	AR	−1.3 [−1.9, −0.7]	±1.4	** −1.56 **	0.2 [−0.0, 0.5]	±0.6	0.10	−1.5 [−1.9, −1.1]	±1.1	** −1.12 **
Free	FE	−7.7 [−9.8, −5.6]	±5.3	** −1.27 **	0.5 [−0.1, 1.0]	±1.3	0.00	−8.2 [−10.0, −6.3]	±4.8	** −1.16 **
	LB	3.0 [1.4, 4.7]	±4.2	0.28	2.5 [0.9, 4.0]	±4.0	0.42	0.6 [−0.1, 1.2]	±1.6	−0.07
	AR	−1.4 [−2.0, −0.8]	±1.5	** −1.66 **	0.0 [−0.3, 0.3]	±0.8	−0.03	−1.5 [−2.0, −1.0]	±1.3	** −1.24 **

FE = flexion extension; LB = lateral bending; AR = axial rotation; Asym. = asymmetric.

**Table 3:** Concordance Correlation Coefficient (CCC) for Range of Motion (ROM) of Thoracolumbar (T12/L1) joint angles in each plane of motion, with 95% Confidence Interval (CI).

Lift	Plane	OMC vs. Xsens	OMC vs. OpenSense	OpenSense vs. Xsens
		CCC [95% CI]	CCC [95% CI]	CCC [95% CI]
Asym.	FE	0.031 [−0.61, 0.65]	0.53 [−0.14, 0.87]	0.039 [−0.61, 0.65]
	LB	−0.16 [−0.72, 0.52]	0.052 [−0.60, 0.66]	−0.048 [−0.66, 0.60]
	AR	−0.0028 [−0.63, 0.63]	−0.40 [−0.82, 0.30]	0.0066 [−0.63, 0.63]
Squat	FE	0.050 [−0.60, 0.66]	0.70 [0.12, 0.92]	0.043 [−0.60, 0.65]
	LB	0.085 [−0.58, 0.68]	−0.35 [−0.80, 0.35]	0.38 [−0.33, 0.82]
	AR	0.028 [−0.61, 0.65]	0.086 [−0.57, 0.68]	0.019 [−0.62, 0.64]
Stoop	FE	0.020 [−0.62, 0.64]	0.33 [−0.37, 0.80]	0.020 [−0.62, 0.64]
	LB	0.10 [−0.56, 0.69]	−0.034 [−0.65, 0.61]	0.18 [−0.51, 0.73]
	AR	0.0072 [−0.63, 0.63]	0.059 [−0.59, 0.66]	0.082 [−0.58, 0.68]
Free	FE	0.054 [−0.60, 0.66]	0.61 [−0.034, 0.90]	0.066 [−0.59, 0.67]
	LB	−0.096 [−0.68, 0.57]	−0.098 [−0.69, 0.57]	0.69 [0.11, 0.92]
	AR	−0.0035 [−0.63, 0.63]	−0.40 [−0.82, 0.31]	0.063 [−0.59, 0.67]

FE = flexion extension; LB = lateral bending; AR = axial rotation; Asym. = asymmetric.

**Table 4:** Lumbosacral root mean squared error (RMSE) and mean absolute error (MAE) between systems across the entire lifting cycle. Values are mean ± SD and in degrees.

Lift	Plane	OMC vs. XSens		OMC vs. OpenSense		OpenSense vs. XSens	
		RMSE	MAE	RMSE	MAE	RMSE	MAE
Asym.	FE	7.9±3.1	6.7±2.8	1.8±0.9	1.5±0.9	8.3±2.1	6.8±1.8
	LB	4.0±0.8	3.1±0.5	0.8±0.3	0.7±0.3	3.9±0.9	3.0±0.6
	AR	3.2±0.9	2.6±0.9	2.3±0.9	1.9±0.9	1.9±0.9	1.6±0.8
Free	FE	7.4±2.8	6.3±2.3	1.7±0.9	1.4±0.8	7.6±2.1	6.3±1.5
	LB	1.7±1.4	1.5±1.1	0.9±0.4	0.8±0.4	1.8±1.8	1.5±1.4
	AR	3.0±2.2	2.7±2.2	1.9±1.0	1.7±1.0	2.2±2.1	2.1±2.1
Squat	FE	6.5±2.2	5.4±2.0	1.6±0.7	1.3±0.6	6.7±1.8	5.5±1.5
	LB	1.8±0.9	1.6±0.7	0.9±0.7	0.9±0.7	2.0±1.4	1.6±1.1
	AR	3.0±1.4	2.8±1.5	2.1±1.1	2.0±1.1	2.3±2.4	2.2±2.4
Stoop	FE	8.5±2.3	7.2±2.0	2.1±1.0	1.7±0.9	8.8±1.6	7.3±1.3
	LB	1.4±0.7	1.2±0.6	0.8±0.3	0.7±0.2	1.4±1.0	1.1±0.8
	AR	2.4±0.8	2.2±0.8	2.1±0.8	1.9±0.8	1.2±0.6	1.1±0.6

FE = flexion extension; LB = lateral bending; AR = axial rotation; Asym. = asymmetric.

**Table 5:** Bland–Altman Agreement: Range of Motion (ROM) of Lumbosacral (L5/S1) joint angles (degrees) in each plane of motion, with 95% Confidence Interval (CI), Limits of Agreement (LoA), and proportional bias slope (β). Bold β indicates significance at α=0.05.

Lift	Plane	OMC vs. Xsens			OMC vs. OpenSense			OpenSense vs. Xsens		
		Bias [95% CI]	LoA	β	Bias [95% CI]	LoA	β	Bias [95% CI]	LoA	β
Asym.	FE	−13.6 [−16.7, −10.4]	±7.9	** −0.88 **	1.8 [0.2, 3.3]	±3.9	−0.07	−15.3 [−17.6, −13.0]	±5.9	** −0.74 **
	LB	−12.8 [−15.1, −10.6]	±5.7	** −1.65 **	−0.4 [−0.8, 0.1]	±1.1	−0.32	−12.4 [−14.5, −10.3]	±5.4	** −1.49 **
	AR	−6.7 [−7.9, −5.5]	±3.1	−0.79	−1.9 [−3.2, −0.5]	±3.4	−0.66	−4.8 [−6.3, −3.2]	±3.9	−0.55
Squat	FE	−12.2 [−14.8, −9.6]	±6.7	** −0.61 **	1.1 [−0.3, 2.5]	±3.5	0.34	−13.3 [−15.7, −11.0]	±6.0	** −0.82 **
	LB	−3.5 [−5.5, −1.6]	±5.0	** −1.97 **	−0.1 [−0.5, 0.3]	±1.0	0.04	−3.5 [−5.4, −1.5]	±5.1	** −2.02 **
	AR	−1.3 [−2.1, −0.6]	±1.9	−0.49	0.1 [−0.7, 1.0]	±2.2	−0.34	−1.5 [−2.1, −0.8]	±1.6	−0.21
Stoop	FE	−14.5 [−17.3, −11.7]	±7.2	−0.50	1.8 [−0.1, 3.8]	±4.9	0.63	−16.3 [−18.3, −14.4]	±5.0	** −0.83 **
	LB	−2.6 [−3.9, −1.3]	±3.4	** −1.46 **	0.1 [−0.4, 0.5]	±1.1	−0.32	−2.7 [−4.2, −1.1]	±4.0	** −1.76 **
	AR	−1.2 [−2.1, −0.3]	±2.3	−0.44	0.6 [−0.4, 1.6]	±2.5	−0.15	−1.8 [−2.8, −0.8]	±2.5	−0.34
Free	FE	−13.3 [−17.6, −9.1]	±10.8	** −0.91 **	1.7 [−0.2, 3.5]	±4.7	0.00	−15.0 [−18.4, −11.7]	±8.5	** −0.81 **
	LB	−3.1 [−5.4, −0.9]	±5.8	** −2.18 **	−0.1 [−0.6, 0.5]	±1.4	** −1.61 **	−3.1 [−5.3, −0.8]	±5.7	** −1.80 **
	AR	−1.4 [−2.5, −0.3]	±2.9	−0.89	0.7 [−0.1, 1.6]	±2.1	−0.51	−2.1 [−3.3, −1.0]	±3.0	−0.40

FE = flexion extension; LB = lateral bending; AR = axial rotation; Asym. = asymmetric.

**Table 6:** Concordance Correlation Coefficient (CCC) for Range of Motion (ROM) of Lumbosacral (L5/S1) joint angles in each plane of motion, with 95% Confidence Interval (CI).

Lift	Plane	OMC vs. Xsens	OMC vs. OpenSense	OpenSense vs. Xsens
		CCC [95% CI]	CCC [95% CI]	CCC [95% CI]
Asym.	FE	0.081 [−0.58, 0.68]	0.53 [−0.14, 0.87]	0.092 [−0.57, 0.68]
	LB	0.0083 [−0.62, 0.63]	0.30 [−0.41, 0.78]	0.014 [−0.62, 0.64]
	AR	0.040 [−0.60, 0.65]	−0.047 [−0.66, 0.60]	0.047 [−0.60, 0.66]
Squat	FE	0.12 [−0.55, 0.70]	0.70 [0.12, 0.92]	0.097 [−0.57, 0.69]
	LB	−0.0044 [−0.63, 0.63]	−0.39 [−0.82, 0.32]	−0.013 [−0.64, 0.62]
	AR	0.33 [−0.37, 0.80]	0.32 [−0.39, 0.79]	0.42 [−0.29, 0.83]
Stoop	FE	0.050 [−0.60, 0.66]	0.33 [−0.37, 0.80]	0.046 [−0.60, 0.66]
	LB	0.10 [−0.56, 0.69]	−0.13 [−0.70, 0.54]	0.016 [−0.62, 0.64]
	AR	0.38 [−0.33, 0.81]	0.24 [−0.46, 0.75]	0.25 [−0.45, 0.76]
Free	FE	0.13 [−0.54, 0.70]	0.61 [−0.034, 0.90]	0.15 [−0.53, 0.71]
	LB	−0.041 [−0.65, 0.60]	−0.12 [−0.70, 0.55]	0.00090 [−0.63, 0.63]
	AR	0.18 [−0.51, 0.73]	0.31 [−0.39, 0.79]	0.17 [−0.52, 0.72]

FE = flexion extension; LB = lateral bending; AR = axial rotation; Asym. = asymmetric.

## Data Availability

The raw data supporting the conclusions of this article will be made available by the authors on request.

## Abbreviations

The following abbreviations are used in this manuscript:

IMU	Inertial measurement unit
OMC	Optical motion capture
MAE	Mean absolute error
RMSE	Root mean squared error
LoA	Limits of agreement
FE	Flexion extension
LB	Lateral bending
AR	Axial rotation
CI	Confidence interval
ROM	Range of motion
DOFs	Degrees of freedom
